# 3D Reconstruction of Space Objects from Multi-Views by a Visible Sensor

**DOI:** 10.3390/s17071689

**Published:** 2017-07-22

**Authors:** Haopeng Zhang, Quanmao Wei, Zhiguo Jiang

**Affiliations:** 1 Image Processing Center, School of Astronautics, Beihang University, Beijing 100191, China; weiqm@buaa.edu.cn; 2 Beijing Key Laboratory of Digital Media, Beihang University, Beijing 100191, China

**Keywords:** space object, 3D structural model, 3D reconstruction, structure from motion, point cloud refinement

## Abstract

In this paper, a novel 3D reconstruction framework is proposed to recover the 3D structural model of a space object from its multi-view images captured by a visible sensor. Given an image sequence, this framework first estimates the relative camera poses and recovers the depths of the surface points by the structure from motion (SFM) method, then the patch-based multi-view stereo (PMVS) algorithm is utilized to generate a dense 3D point cloud. To resolve the wrong matches arising from the symmetric structure and repeated textures of space objects, a new strategy is introduced, in which images are added to SFM in imaging order. Meanwhile, a refining process exploiting the structural prior knowledge that most sub-components of artificial space objects are composed of basic geometric shapes is proposed and applied to the recovered point cloud. The proposed reconstruction framework is tested on both simulated image datasets and real image datasets. Experimental results illustrate that the recovered point cloud models of space objects are accurate and have a complete coverage of the surface. Moreover, outliers and points with severe noise are effectively filtered out by the refinement, resulting in an distinct improvement of the structure and visualization of the recovered points.

## 1. Introduction

Detecting, tracking, cataloging and identifying man-made objects orbiting Earth comprise one of the fundamental requirements of space surveillance [1]. More and more states are pursuing such space surveillance systems to develop their space surveillance capabilities [2]. In recent years, space-based surveillance systems [3,4,5] and high-performance optical imaging sensors have been rapidly developed. Visible sensors used by space-based surveillance systems can avoid the impact of atmospheric turbulence, which severely influences traditional ground-based systems. Meanwhile, space-based surveillance systems can also provide higher spatial resolution image data at a closer distance.

Object categorization, recognition and pose estimation of space objects are the main tasks in the fields of space exploitation and surveillance. In terms of pose estimation, Zhang et al. [6] presented a vision-based method. Zhang et al. [7] proposed an improved pose estimation algorithm based on 2D-3D correspondences between the input image and the 3D model of space objects. For space object recognition, different features and clustering algorithms were studied [8,9,10,11]. To handle the tasks of both recognition and pose estimation in one vision-based framework, methods based on kernel regression [12] and homeomorphic manifold analysis [13] were proposed. However, all of these methods require, directly or indirectly, the prior 3D models of space objects. For example, the method of [7] needs the 3D model to provide 2D-3D correspondences, and those of [6,8,9,10,11,12,13] need images from multiple or full viewpoints, which are difficult to access without prior models of the targets. Thus, obtaining 3D models is important for vision-based tasks in space surveillance.

In this paper, we address the problem of recovering space object models by reconstruction from images captured by space-based visible sensors. Compared to model generation by laser scanning, image-based 3D reconstruction is economical and convenient. Moreover, it is also a non-contact and noninvasive measurement, which is suitable for space missions, especially for non-cooperative targets. On the one hand, the recovered space object models and full-viewpoint images generated with the recovered models are helpful for the recognition and pose estimation. Furthermore, the recovered 3D models could lead to a better result for automatic measurement and pose estimation of the sub-components, as these 3D models do not contain the perspective projection, which must be taken into account when handling two-dimensional images. On the other hand, the recovered 3D models have a great application value for space missions, such as autonomous rendezvous and docking, collision avoidance and on-orbit self-serving. Moreover, the recovered models could further reveal the functional characteristics of space objects, which are significant for space situational awareness.

Multiple view geometry has developed rapidly since the 1990s [14], and many image-based reconstruction systems have been proposed [15,16,17,18,19,20,21,22,23]. However, there are few works that focus on the 3D structural model reconstruction of space objects, which is indeed the goal of this paper. This paper is a follow-up to the work in [24]. In this paper, traditional multi-view-image-based reconstruction pipelines are applied, including image feature point extracting and matching, sparse reconstruction by the structure from motion (SFM) method [14,22,23] and dense reconstruction by the patch-based multi-view stereo (PMVS) algorithm [25]. A special modification of the strategy of adding new images during SFM is introduced to resolve the wrong matches that arise from the symmetric structure and the repeating textures of space objects. Additionally, a refinement method exploiting the structural characteristic that most sub-components of space objects are made out of basic geometric shapes is proposed to improve the visualization of the recovered point cloud model. The experimental results on both simulated image datasets and real image datasets have demonstrated the reconstruction ability and accuracy of this framework.

This paper is organized as follows. A brief introduction of the framework is given in Section 2. Details of the reconstruction and point cloud refining method are described in Section 3 and Section 4, respectively. Experimental results are shown in Section 5. At last, Section 6 provides the conclusion.

## 2. Overview of the Framework

**Figure 1:**
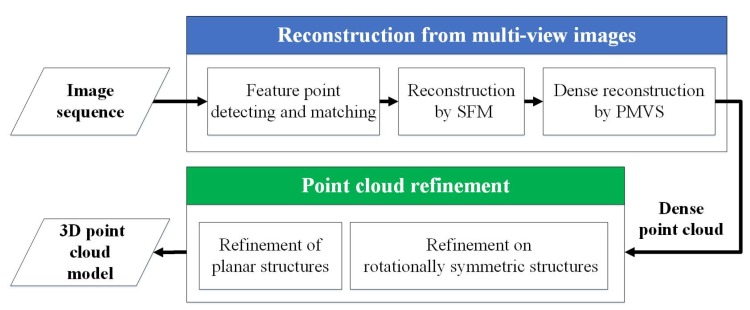
Framework for the reconstruction of space objects. PMVS, patch-based multi-view stereo.

The reconstruction framework for space objects consists of two parts, i.e., reconstruction of the 3D structural model from multi-view images and refinement of the reconstructed point cloud model, as illustrated in Figure 1.

The theoretical principles of 3D reconstruction are well defined, and general reconstruction pipelines, especially the SFM, are also widely used. Therefore, to recover the 3D structural model from multi-view images, our framework employs a general feature point-based multi-view reconstruction procedure, which consists of three steps:

(a) Detect feature points in each image and match the feature points between each pair of images.
(b) Estimate the relative poses of each view and recover the depths of the detected points, i.e., the SFM step.
(c) Taking the results of SFM as inputs, use the PMVS algorithm [25] to generate a dense 3D point cloud.

Due to the structure symmetry and repeated textures of space objects, there might be enormous error matches between symmetrical viewpoints, which meet the geometrical conditions while disagreeing with the actual imaging configurations. These error matches would further lead to overlap and failure of the reconstruction. To handle such mismatches, a special modification is made on the strategy of adding new images during the SFM step. This paper assumes that the space objects are mainly composed of planar solar wings and a cuboid/cylinder main body, and the datasets used in our experiments basically satisfy this assumption. Small and thin functional/non-functional components, such as antennas, imaging sensors, nozzles and trusses, are all ignored (actually treated as outliers) in our refinement processing. However, such simplified general models are still sufficient for most space applications.

## 3. 3D Reconstruction from Multi-View Images

In this paper, a general linear pinhole camera model is adopted. The intrinsic parameter matrix K of the camera is modeled as:

(1) $$K=\left[\begin{array}{ccc} f & 0 & c_{x}\\ 0 & f & c_{y}\\ 0 & 0 & 1 \end{array}\right],$$

where *f* denotes the focal length in pixels, $\left(c_{x},c_{y}\right)$ represents the image coordinate of the principal point, i.e., the point where the principal axis of the camera intersects the image plane. Since there is little distortion in our simulated images, a single ideal camera with a fixed focal length is adopted rather than a more complex distorted one. Due to the development of calibration methods, it is easy to calibrate the intrinsic parameters with high precision. Thus, a given intrinsic parameter matrix K, which could be calibrated in advance, is required to simplify the problem.

### 3.1. Feature Point Detecting and Matching

The scale-invariant feature transform (SIFT) keypoints [26] are extracted for each image as feature points because of their stability and invariance to image transformations. In addition to the keypoint locations, the SIFT detector also provides a 128-dimensional local descriptor for each keypoint, which can be used for the primary match with an approximate nearest neighbor search method. A point ${}^{m}p_{i}$ (the *i*-th point in the *m*-th image) in image $F_{m}$ is matched to a point ${}^{n}p_{j}$ (the *j*-th point in the *n*-th image) in image $F_{n}$ when two conditions are satisfied: (a) among all of the feature points in $F_{n}$, the descriptor of ${}^{n}p_{j}$ has the closest distance (e.g., the Euclidean distance) $d_{1st}$ to that of ${}^{m}p_{i}$; (b) $d_{1st}$ is much smaller than the second closest distance $d_{2nd}$, i.e., $d_{1st}<\lambda d_{2nd}$, 0<λ<1. Then, for point matches $\left\{{}^{m}p_{i},{}^{n}p_{j}\right\}$ between image pair $F_{m}$ and $F_{n}$, to remove the outliers, the random sample consensus (RANSAC) algorithm [27] is used, in which the epipolar constraint is employed, i.e.,

(2) $${}^{m}p_{i}E\;{}^{n}p_{j}=0,$$

where ${}^{m}p_{i}$ and ${}^{n}p_{j}$ denote the homogeneous normalized coordinate vectors of a point pair between image frame $F_{m}$ and $F_{n}$. Here, the normalization means conversion from image coordinate to camera coordinate, i.e., $p\equiv \hat{p}=K^{-1}{\left[\begin{array}{ccc} x & y & 1 \end{array}\right]}^{T}$, where $\left(x,y\right)$ is the image coordinate. $E={\left[t\right]}_{\times }R$ is the essential matrix, which can be parameterized by rotation matrix R and translation vector t, and ${\left[•\right]}_{\times }$ denotes the skew-symmetric matrix, such that ${\left[a\right]}_{\times }x=a\times x$.

The essential matrix E, with a non-zero scale factor, is simultaneously estimated by a robust method, such as the eight-point algorithm [28], during the RANSAC iteration. A more accurate estimation of E can be achieved by non-linear refinement after the iteration.

### 3.2. Structure from Motion

Structure from motion begins with the relative camera pose estimation of an initial image pair. Such an initial image pair is selected with certain rules, for instance the image pair that has the largest number of point matches and a large baseline, as proposed in [22]. Assume $F_{0}$ and $F_{1}$ are the initial image pair. Since the essential matrix $E_{1}$ between $F_{0}$ and $F_{1}$ has already been found in Section 3.1, the rotation matrix $R_{1}$ and translation vector $t_{1}$ of $F_{1}$, referring to the camera coordinate of $F_{0}$, can be further extracted from $E_{1}$ by SVD-based techniques. Then, the 3D locations of the matched point pairs between $F_{0}$ and $F_{1}$ can be recovered by triangulation, yielding an initial 3D point set $M_{1}$. To refine both the estimated camera pose (i.e., the rotation matrix R and translation vector t) and the recovered 3D point set jointly, bundle adjustment is used. The goal of bundle adjustment is to minimize the reprojection errors through optimizing the positions of both the cameras and the observed points, i.e.,

(3) $$\min_{P_{j}\in M_{n},R_{n},t_{n}}\sum_{i=0}^{n}\sum_{j=0}^{m_{i}-1}∥{}^{i}p_{j}-\left[R_{i}|t_{i}\right]P_{j}∥,$$

where $M_{n}=\left\{P_{j}\right\}$ is the last updated 3D point set with a size of $m_{i}$. $R_{i}$ and $t_{i}$ are the rotation matrix and translation vector, which indicate the camera pose of image $F_{i}$. ${}^{i}p_{j}$ is the projection point of the recovered 3D point $P_{j}$ in image $F_{i}$. Here, n=1 for the initial pair.

Next, the image that observes the largest number of recovered points in $M_{n-1}$ is added into the *n*-th iteration. Let $M_{n-1}$ be the recovered 3D point set after adding the (n−1)-th image $F_{n-1}$. The rotation matrix $R_{n}$ and translation vector $t_{n}$ are then estimated from the 2D-3D point correspondences between $F_{n}$ and $M_{n-1}$. Next, point matches between the new added image $F_{n}$ and all of the previous images $F_{m}\left(m=0,1,\cdots ,n-1\right)$, but having no corresponding point in $M_{n-1}$, are recovered, resulting in the increase of size from $M_{n-1}$ to $M_{n}$, i.e., a more complete reconstructed structure. At last, the bundle adjustment is applied to optimize $R_{n}$, $t_{n}$ and $M_{n}$. This procedure is repeated until there is no image remaining.

**Figure 2:**
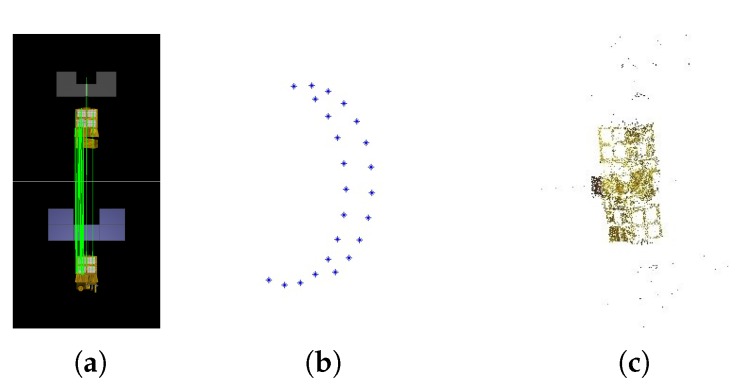
Incorrect reconstruction caused by symmetric structures and repeated textures. (**a**) Matches; (**b**) poses; (**c**) point cloud.

**Figure 3:**
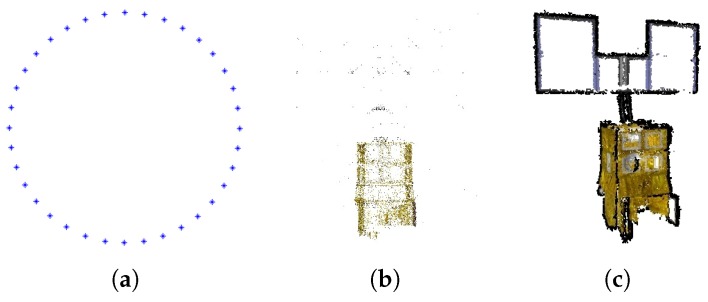
Correct reconstruction with modified image adding strategy. (**a**) Poses; (**b**) point cloud (sparse); (**c**) point cloud (dense).

Artificial space objects, in common with most industrial products, are designed with symmetric structures and have repeating textures. These characteristics might cause point matches between images that are seen from opposing viewpoints, as shown in Figure 2a. Then, a wrong camera pose would be estimated from such unexpected matches and further influence the rest of the estimations of camera poses. As shown in Figure 2b, the recovered camera viewpoints are approximately located on a half of circle, while in fact, the test images are taken from viewpoints uniformly located on a full circle. The final point cloud recovered is incorrect, as shown in Figure 2c. To solve this problem, we modify the original strategy where only the number of point correspondences is considered. Since the inputs in our cases are image sequences, i.e., the images are well sorted in imaging order, the overlaps between neighboring images should ensure enough point matches. Therefore, to avoid the unexpected matches between images from opposing viewpoints and ensure enough correct matches between the newly-added image and recovered points, as well, our modified strategy is to add the images only in the order that they are captured. The correct reconstruction result with our modified image adding strategy is shown in Figure 3, where both the viewpoints and structural model are correctly recovered.

### 3.3. Dense Reconstruction

Through SFM, we can estimate the relative camera pose of each image and recover the 3D location of feature points, but such reconstruction of the feature points is not enough to reveal the structure of the target due to the poor surface coverage. Therefore, to improve the reconstruction coverage, the PMVS algorithm [25] is employed. The space object images, in which the backgrounds are not cluttered and the object is usually prominent, are fine inputs for PMVS, as it could be relatively straightforward to extract the contours. After the iterations of the matching, expanding and filtering procedure, PMVS can output a dense set of rectangular patches covering the object surfaces that are visible in the input images. Each patch is defined by a combination of a center point and a normal vector.

## 4. Point Cloud Refinement

The main body of the space object might be a cuboid or symmetric with respect to its major axis, and the solar panels are always flat and could be treated as rectangles when ignoring their thickness. With this structural prior information that most sub-components are in basic geometric shapes, refinements for planes and rotationally-symmetric structures are introduced respectively. Although the precise refinement operations for these two structures are various, they all follow the same procedure. Specifically, the basic geometric structure is first detected, then points belonging to this structure are discriminated, and finally, an adjustment is done to each point.

### 4.1. Refinement for Planes

**Figure 4:**
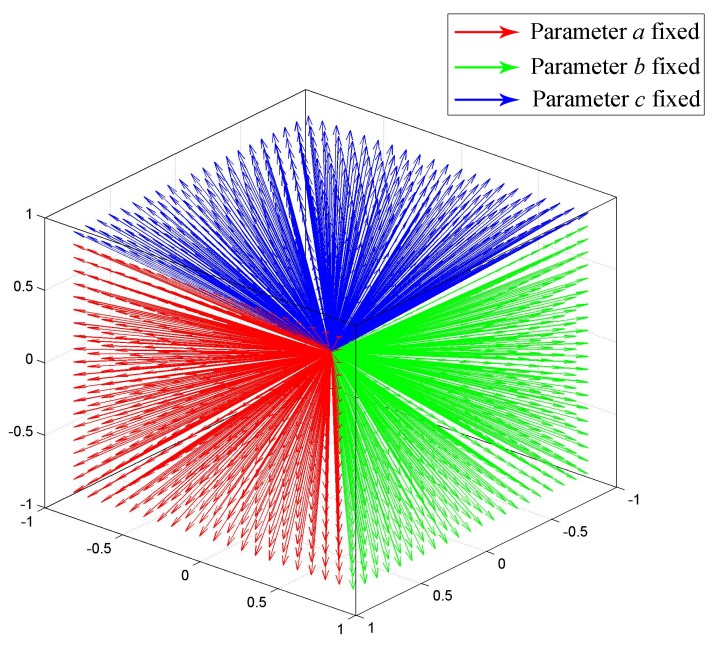
Illustration of normal vectors. All of the possible normal vectors of Π are approximately uniformly distributed in the first octant. Here, the discretized resolution of the plane parameters is 0.1.

The planes are first detected by Hough transform in 3D space. Assume Π is some 3D plane to be detected, then Π can be formulated as ax+by+cz+d=0, where $\left(x,y,z,1\right)\in \Pi$ is the point on Π in homogeneous format and $\left(a,b,c,d\right)$ is the parameter that defines Π. This formulation can also be treated as follows: a point $\left(a,b,c,d\right)$ is on a plane defined by $\left(x,y,z,1\right)$, due to the dual relation between the plane parameter and point coordinate. Given series points in Π, the parameter $\left(a,b,c,d\right)$ defining Π then can be found as an intersection point of the planes that are defined by these given points. Since (a,b,c,d) is homogeneous, any parameter (λa,λb,λc,λd) (λ≠0) refers to the same plane. To ensure uniqueness, *a*, *b* and *c* are fixed to one separately in three separate Hough detections, and the other three parameters are discretized and limited in range $\left[-1,1\right]$. Discretization with resolution 0.1 is illustrated as Figure 4, where all of the possible normal vectors are approximately uniformly distributed. The plane parameters of Π then correspond with a discretized point in the parameters space, and the final plane is picked from three candidates obtained by the three Hough detections.

After plane Π is detected, points that belong to the plane structure Π are then discriminated. A point *p* is discriminated to be on Π when: (a) *p* is near Π; and (b) the normal vector angle between *p* and Π is small. Considering that different planar components might be located on the same plane, e.g, two symmetrically-assembled solar wings that face in the same direction, the region growing algorithm is employed to distinguish such different components. At last, each point discriminated to be on Π is moved to its projection on Π, and the normal vectors of these points are set to be parallel with the normal vector of Π.

The whole procedure of the plane refinement is shown in Algorithm 1. Multiple planes are iteratively detected, until the last detected plane is too small or the number of detected planes exceeds a expected maximum $Max_{np}$ ($Max_{np}=8$ in our experiments).
**Algorithm 1:** Refinement for planes.
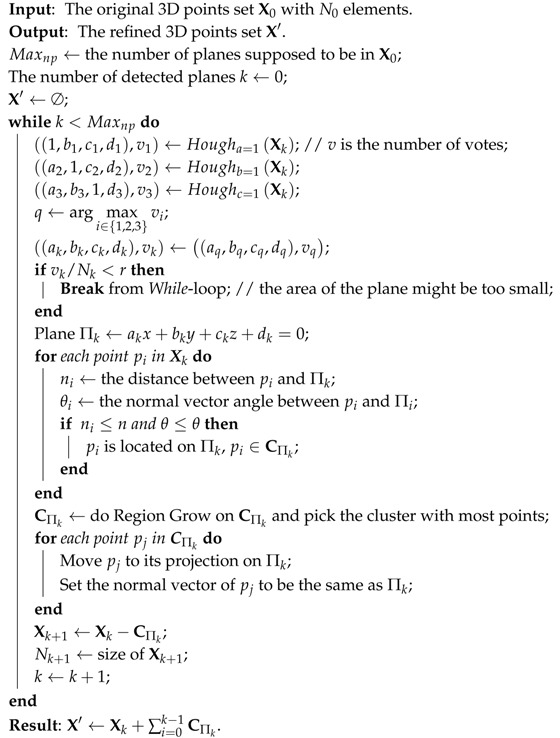



### 4.2. Refinement for Rotationally-Symmetric Structures

Parameterizing a cylinder requires more parameters than a simple plane; this increase of parameter count would result in a geometrical growth of calculation in Hough transformation. Moreover, rotationally-symmetric structures are not limited to the cylinder, namely the parametrization of rotationally-symmetric structures could be complicated and vary with a specific structure. Considering the common characteristic of all rotationally-symmetric structures, consisting of the surface normal always intersecting the symmetry axis, the detection of such rotationally-symmetric structure could be converted into the detection of the symmetric axis from those intersections. The 3D space is first discretized to voxels at a suitable resolution. Next, the voxels are voted based on the surface normals passing through them. Allowing for the existence of intersections of non-surface normals and the error of surface normals, only voxels with enough votes are accepted as the right intersections of surface normals. Then, principal component analysis (PCA) is used to robustly estimate the symmetry axis from these intersections, and the votes are used as weights.

After finding the symmetry axis, the surface points are discriminated preliminarily by the rule that a point is a surface point if the distance between its normal and the symmetry axis is short enough. Then, the surface points are refined, and during the refinement, these points are verified again. First, the space is re-divided into layers with a thickness of Δd along the symmetry axis. Then, the mean radius $R_{i}$ is calculated for each layer $L_{i}$. Referring to $R_{i}$, points too far away from or too near to the symmetry axis are removed from surface points, and $R_{i}$ is recalculated after this verification. At last, each surface point in $L_{i}$ is moved along the radius direction to keep a distance of $R_{i}$ from the symmetry axis, and the normals of these points are set so that they will exactly intersect the symmetry axis at the mean position of the original intersections.

**Figure 5:**
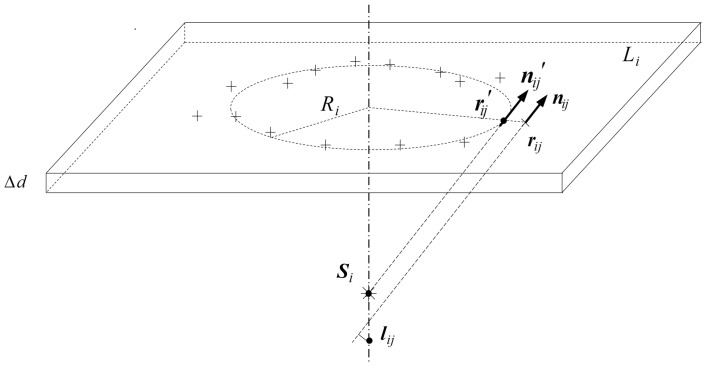
Illustration of point adjustment for rotationally-symmetric structures. For layer $L_{i}$, move the point at $r_{ij}$ along the radius direction to $r_{ij}^{\prime }$, and set its normal as $n_{ij}^{\prime }=S_{i}-r_{ij}^{\prime }$, where $S_{i}$ is the trimmed mean location of {$l_{ij}$}.

The whole refinement procedure for rotationally-symmetric structures is shown in Algorithm 2, and the adjustment of the surface points is shown in Figure 5.
**Algorithm 2:** Refinement for rotationally-symmetric structures.
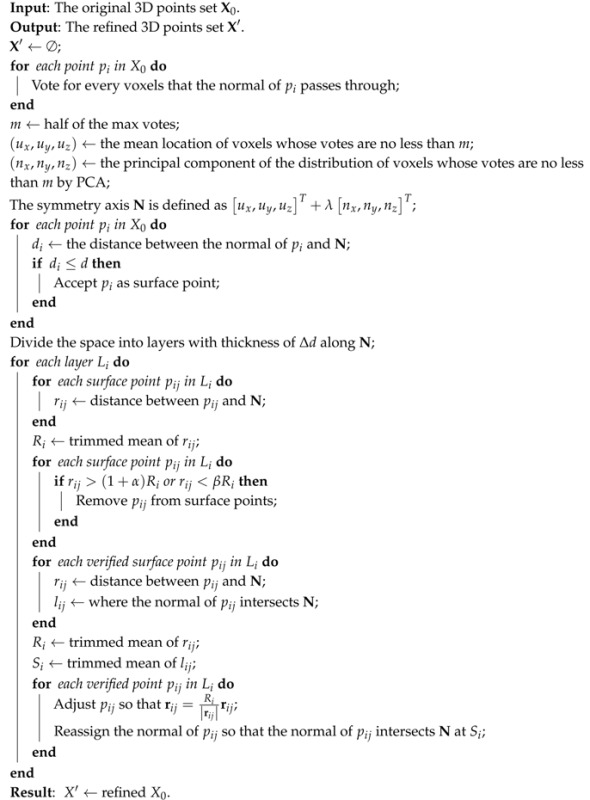



## 5. Experiments and Analyses

In this section, the proposed space object reconstruction framework is tested on the datasets in Section 5.1. Note that the reconstruction procedure in our framework is implemented based on Bundler [22], where the image addition mechanism is modified. To quantitatively verify the feasibility and accuracy of the refinement method, experiments are conducted on both simulated images and real images.

### 5.1. Data Collection

**Figure 6:**
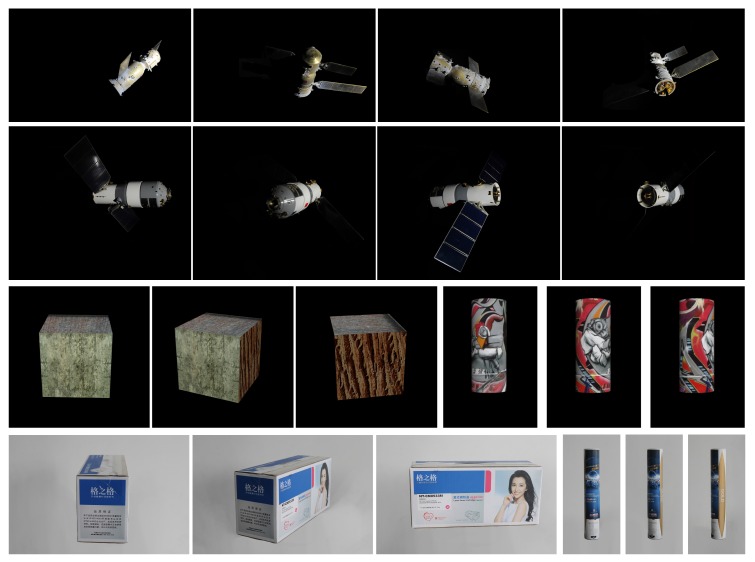
Samples of image data used in our experiments. From top to bottom: image samples of Shenzhou-6 (**the first row**) and Tiangong-1 (**the second row**), rendered images of two 3D CAD models (**the third row**) and real images taken from two actual packages (**the fourth row**).

The image data used in the experiments include the following image sets:

1. Images of spacecraft Shenzhou-6 and Tiangong-1 from the ground imaging simulation experiment. In the ground imaging simulation experiment, a dark room covered with cloth absorbing light inside and cloth reflecting light outside is established to simulate the space environment. Parallel light is used to simulate the sun light. Camera is fixed pointing at the scaled model of the space object, which is put on a one-degree-of-freedom turntable and rotated along with the turntable. One frame is taken every 10° of rotation of the turntable. The supporter and turntable in images are erased later, as shown in Figure 6.
2. Rendering images of CAD models of a box and a cylinder: The virtual camera is fixed pointing at the model, and the geometric models are attached with different textures. One hundred frames are rendered during a 360° rotation of the model, i.e., one frame is taken every 3.6° the model rotates, as shown in Figure 6.
3. Real images taken from two packages separately: The chosen packages are a packing box of a printer cartridge and a packing canister for badminton, respectively, representing a standard box and a standard cylinder. The package is put on the one-degree-of-freedom turntable and rotated along with it, and the camera is fixed pointing at the package. One image is taken every 2° of rotation. The supporter and turntable in the images are erased later, as well, as shown in Figure 6.

### 5.2. Parameter Determination for Point Cloud Refinement

In the point cloud refinement, several thresholds are used, including distance thresholds and angle thresholds. Since the reconstructed 3D point cloud model is up to a scale factor in size, the distance thresholds are relative as well. Thus, to determine such distance thresholds, factor *a*, which can be regarded as a measurement unit, is first defined based on the overall size of the reconstructed point cloud. In our experiments, *a* is adaptively defined as 1% of $D_{3}$, where $D_{3}$ is the dimension along the last eigenvector ($\lambda_{1}\geq \lambda_{2}\geq \lambda_{3}$) of the covariance tensor of these 3D points. The discretization resolutions of the 3D space for the detection of both planes and rotationally-symmetric structures are *a*. A set of *a*-based thresholds that can contribute to a fine performance are determined by experience, as listed in Table 1.

**Figure 7:**
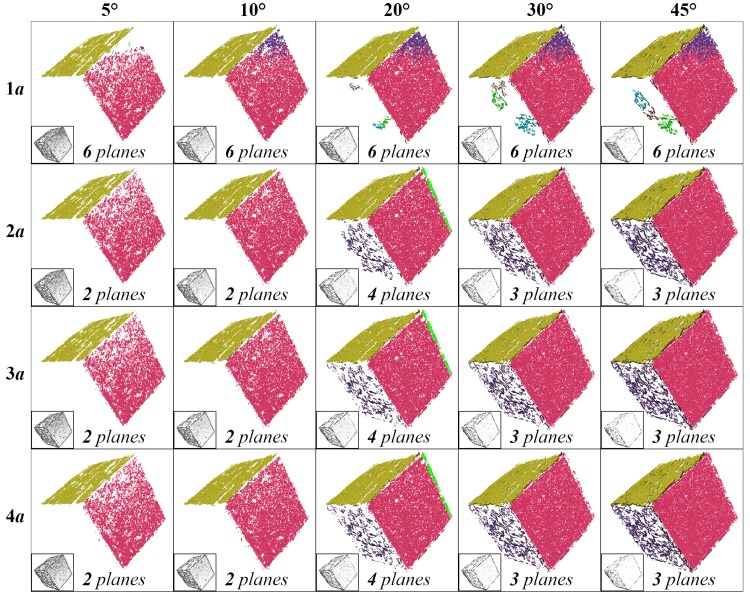
Results of plane detection with different verification thresholds *n* (rows) and θ (columns). $Max_{np}$ in Algorithm 1 is six. Multiple planes are detected with different colors; point clouds at the lower left corner are the points filtered out.

**Figure 8:**
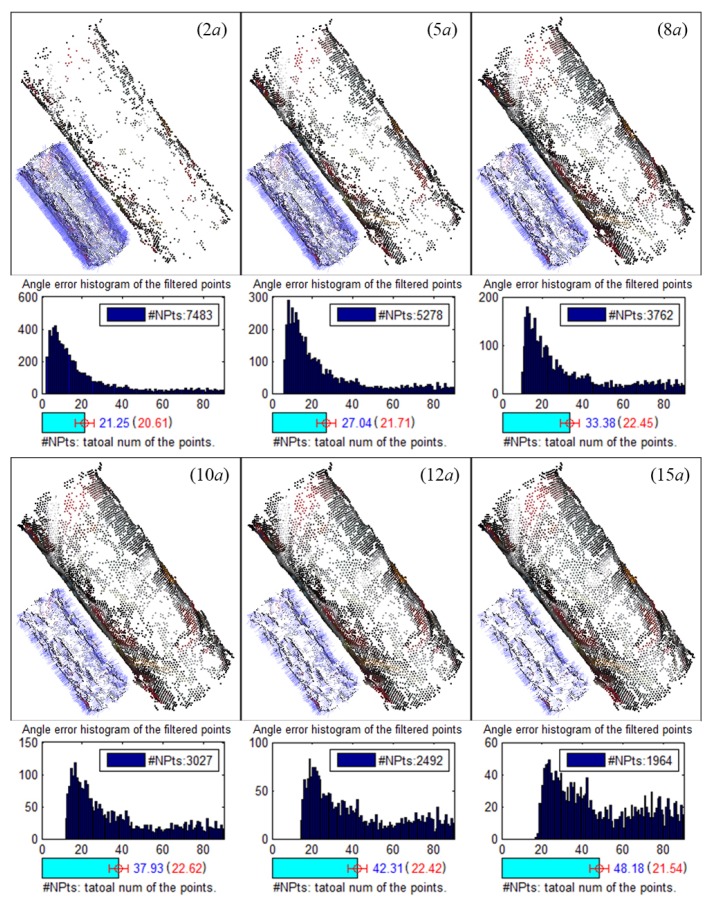
Results of rotationally-symmetric structure detection with different verification threshold *d*. Point clouds at the lower left corner, which are rendered with normals, are the points filtered out. The bar chart below the point cloud shows the histogram of the angle error of these filtered points, along with a horizontal bar indicating the average and RMS.

Among these threshold parameters, the verification thresholds, i.e., *n* and θ for planes, and *d* for rotationally-symmetric structures, are the primary factors that affect the results, since they are used to decide whether a point is a surface point. Experiments are conducted based on the rendered images of CAD models to evaluate the influence of these verification thresholds. Results of plane detection with different verification thresholds *n* and θ are shown in Figure 7. Small *n* has little robustness to the distance deviation and is more likely to result in repeated detection of the same plane, while small θ has little robustness to the normal deviation. Thus, a moderate combination of n=2a and θ = 30° is used. Figure 8 shows the results of rotationally-symmetric structure detection with different verification thresholds *d*. Similarly, small *d* has little robustness to the normal deviation, while large *d* would abate the filtering effects. Thus, an appropriate small d=10a is finally used.

### 5.3. Reconstruction of Objects with Basic Geometry

**Figure 9:**
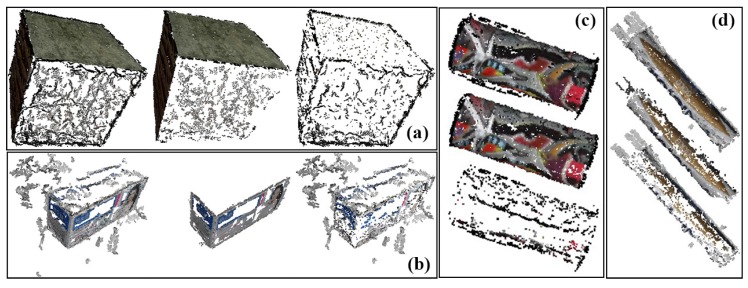
Results of reconstruction and refinement for the box model (**a**), the packing box (**b**), the cylinder model (**c**) and the packing canister (**d**). From left to right (top to bottom): the recovered point cloud, point cloud after being refined and points filtered by refinement.

The results of reconstruction and refinement for the box model and packing box are displayed in Figure 9a,b, where the faces that are visible in the input images are recovered, and the points that have a greater error are filtered out by refinement. Note that, since the simulated images lack good views of the upper face (i.e., viewing above the model), the upper faces of both the box model and packing box are relatively poorly reconstructed. Meanwhile, uniform areas of the packing box cannot be recovered because no stable feature points can be extracted in such areas. Quantitative analysis is shown in Table 2, where the number of recovered points is counted, and the normal vectors of surfaces are calculated, as well. Angles between the normal vectors are {90.6°, 90.6°, 90.5°} and {89.1°}, which are close to the ground truth of 90°.

**Figure 10:**
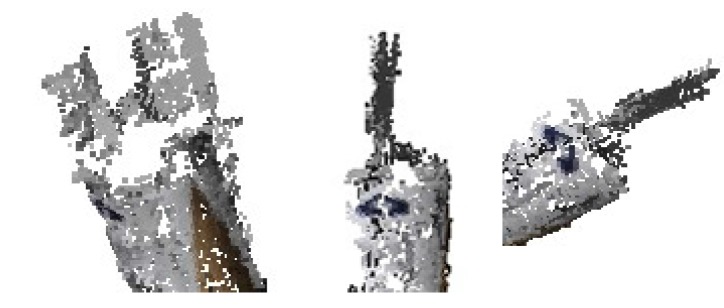
Incorrect reconstruction caused by the erasure of the supporter and turntable.

**Figure 11:**
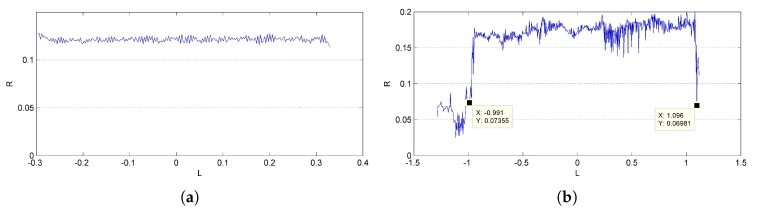
Outline of the reconstructed point cloud for (**a**) cylinder model and (**b**) packing canister.

Results of reconstruction and refinement for the cylinder model and packing canister are displayed in Figure 9c,d. An incorrect recovered part can be found at one end of the packing canister, as shown in Figure 10, that is exactly where the supporter and turntable are erased. The ratio of the length to the radius is calculated for quantitative analysis. The length of the cylinder is directly measured from the outline of the reconstructed points, which is generated with the mean radius *R* in each layer along the symmetry axis *L*, as shown in Figure 11. The mean value of the outlines is calculated as the final radius. The final measurement results are shown in Table 3; deviations from their ground truths are all less than 5%.

### 5.4. Reconstruction of Space Object

**Figure 12:**
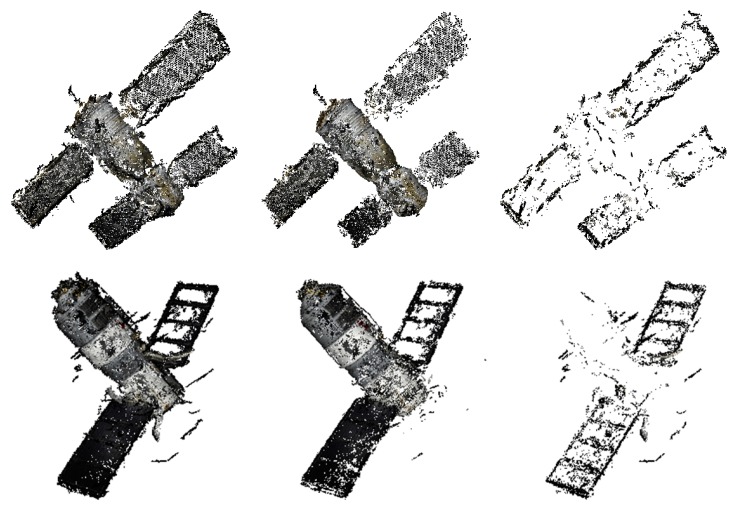
Results of reconstruction and refinement for Shenzhou-6 and Tiangong-1. From left to right: the recovered point cloud, the point cloud after being refined and points filtered by refinement.

**Figure 13:**
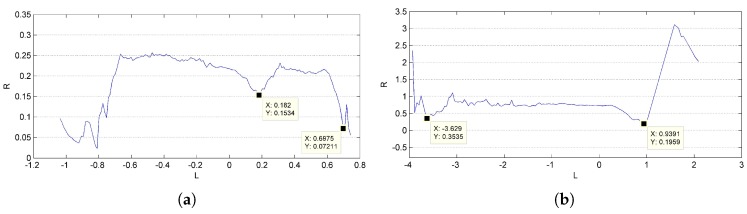
Outline of the reconstructed point cloud for (**a**) Shenzhou-6 and (**b**) Tiangong-1.

The results of reconstruction and refinement for Shenzhou-6 and Tiangong-1 are displayed in Figure 12. The 3D point cloud structures of both Shenzhou-6 and Tiangong-1 are well recovered; the outliers and points that are recovered with severe errors in position and direction are filtered after refinement. Highlights, mirror image and darkness might appear in the same area of the object as the view point changes. This might result in incomplete reconstruction due to not enough stable matches, such as the left solar wing of Tiangong-1 shown in Figure 12. The outlines of the recovered objects are shown in Figure 13, and the results of relative size measurement are shown in Table 3. Since Shenzhou-6 has an orbital capsule, a re-entry capsule and a propelling capsule, which have different radii, only the length-to-radius ratio of the orbital capsule is estimated. The deviations are still very small.

## 6. Conclusions

In this paper, we proposed a reconstruction framework for recovering the structure models of space objects using a visible sensor. Given multi-view images of the target object, which could be captured by a visible sensor on space-based surveillance systems, our framework can recover a 3D point cloud model of the target. Such a model can be used to generate full-viewpoint images of the target and is helpful for further estimation and recognition studies. Furthermore, the reconstructed model has an important practical value, as it can be applied to space missions, such as autonomous rendezvous and docking, collision avoidance and on-orbit self-serving. To resolve the incorrect reconstruction, which is caused by the symmetric structure and repeated textures of space objects, we modify the SFM procedure to avoid the unexpected point matches. Meanwhile, a point cloud refinement utilizing the structural prior is introduced to improve the visualization. Experimental results demonstrate that the proposed reconstruction framework can effectively recover a point cloud model of the space object with both a complete coverage and a fine accuracy, and the visualization of the recovered model can be also obviously improved after refinement. In the future, further performance evaluation and improvement will be made for the consideration of the degrading factors, such as noise and blur caused by relative orbital motion. Automatic measurement and recognition of the key components in the reconstructed point cloud, e.g., solar wings, are also topics worth researching.

## Figures and Tables

**Table 1:** Definition of thresholds used in point cloud refinement.

Geometry	Thresholds Definition		Value
Plane	*r*	Percentage threshold to decide if the proposal plane is big enough to be a plane component.	0.015~0.02
	*n*	Distance threshold to decide if a point is on the plane.	2a
	θ	Angle threshold to decide if a point fits the plane.	30°
Rotationally-Symmetric Structure	*m*	Count threshold to decide if the proposal intersection is a intersection of the surface normals.	Max/2
	*d*	Distance threshold between the normal a point and the symmetry axis to decide if the point is a surface point.	10a
	Δd	The thickness resolution of layers divided along the axis.	a/2
	α	The max radius (1+α)R for a surface point in consideration of errors.	0.5
	β	The min radius βR for a surface point in consideration of errors.	0.2

**Table 2:** Analysis for reconstruction of box objects.

Object	$N_{1}$ *	$N_{2}$ **	Normal Vector of Surfaces
Box Model	41,337	32,761	(0.09,0,1)
			(1,−0.01,−0.1)
			(0,1,−0.01)
Packing Box	27,173	15,266	(1,−0.04,−0.94)
			(0.97,−0.01,1)

* Number of recovered point clouds; ** Number of point clouds after being refined.

**Table 3:** Relative size estimation for space objects.

Object	Length	Radius	λ *	$\lambda_{\mathrm{GT}}$ **	Deviation (%)
Cylinder Model	0.62	0.12	2.58	2.5	3.2
Packing Canister	2.09	0.17	6.15	5.90	4.2
Shenzhou-6	0.52	0.21	1.238	1.24	0.1
Tiangong-1	4.57	0.75	3.047	3.10	1.7

* Ratio of the length to the radius; ** Ground truth of the ratio of the length to the radius.
